# 

**Published:** 1919-06-14

**Authors:** 


### THE COLLEGE OF NURSING (Limited by Guarantee).

### Annual Meeting and Conference, Manchester, June 18 and 19.

**ANNUAL MEETING, Wednesday, June 18, 3 p.m.**

The Hon. Sir Arthur Stanley, G.B.E., in the chair.

**CONFERENCE, Thursday, June 19, 3 p.m.**

Sir Henry Miers, Vice-Chancellor of the Manchester University, and Sir Cooper Perry, M.D., Vice-Chancellor of the London University and Hon. Secretary of the College, in the chair.

Both these gatherings, by the kind permission of the Vice-Chancellor, will be held in the Chemistry Theatre of the University, Oxford Road, Manchester.

**Subjects to be Discussed at Conference.**

The Conference is to be divided into two parts:—

Part I.: "The Further Development of the Usefulness of Local Centres."

Miss Brown, R.R.C., Matron, Royal Victoria Infirmary, Newcastle-on-Tyne; Miss Milne, Sister, South Manchester Hospital, West Didsbury.

Part II.: "The Ideals and Ethics of Nursing."

Miss Gill, R.R.C., Royal Infirmary, Edinburgh; Miss Lloyd Still, C.B.E., R.R.C., St. Thomas's Hospital, London; the Rev. W. S. Swayne B.D., Dean of Manchester.

**SOCIAL FIXTURES.**

**Wednesday, June 18, 7.30 p.m.**

Reception by the Lord Mayor and Lady Mayoress in the Town Hall.

**Thursday, June 19, 11 a.m.**

A visit to one of the following places: (1) The Royal Infirmary; (2) a cotton mill; (3) Chetham College; (4) the Aerodrome.

**Anyone wishing for hospitality or for further particulars regarding the above, may obtain the same from the Hon. Secretary, College Conference, Royal Infirmary, Manchester.**

THE LONDON CENTRE'S CLUB FOR NURSES.

AN American Tea is to be held to raise funds for the Club. Miss Rogers, Carnforth Lodge, Queen Street, Hammersmith, will be "At Home" on Saturday, June 21, from 3.30 P.M. to 7 P.M., to all members of the College and their friends. Each visitor is asked to bring a gift, value at least 1s., and also to purchase something from the stall. Entrance and tea, 1s. 'Buses to Hammersmith Broadway—9, 11, 27, 33, 73, 74; two minutes from Hammersmith Tube, District, or Metropolitan Stations. R.S.V.P. before June 16.

Miss Montgomery will be "At Home" to all members of the Club at 7 Henrietta Street, W., on Friday, June 13, 7 to 9 P.M.

**Matrons' and Sisters' Appointments and other news unavoidably held over.**

